# Microfracture-coagulation for the real robotic liver parenchymal transection

**DOI:** 10.1007/s11701-024-01842-9

**Published:** 2024-02-29

**Authors:** Jordi Navinés-López, Fernando Pardo Aranda, Manel Cremades Pérez, Francisco Espin Álvarez, Alba Zárate Pinedo, Esteban Cugat Andorrà

**Affiliations:** Universitat Autònoma de Barcelona, Hospital Universitari Germans Trias i Pujol de Badalona, HPB unit, Badalona Barcelona, Spain

**Keywords:** Robotic liver surgery, Parenchyma liver transection, Microfracture-coagulation, Real robotic approach

## Abstract

The use of the robotic approach in liver surgery is exponentially increasing. Although technically the robot introduces several innovative features, the instruments linked with the traditional laparoscopic approach for the liver parenchymal transection are not available, which may result in multiple technical variants that may bias the comparative analysis between the different series worldwide. A real robotic approach, minimally efficient for the liver parenchymal transection, with no requirement of external tool, available for the already existing platforms, and applicable to any type of liver resection, counting on the selective use of the plugged bipolar forceps and the monopolar scissors, or “microfracture-coagulation” (MFC) transection method, is described in detail. The relevant aspects of the technique, its indications and methodological basis are discussed.

## Introduction

The increase of the robotic liver surgery (RLS) approach is exponential, at the expense of a decrease in the laparoscopic liver surgery (LLS) approach, which since 2018 has experienced a decline in the number of resections worldwide [1]. This increase has been parallel to a very notable growth of the companies dedicated to the manufacture and development of surgical robots, with high revenues only in 2022, as disclosed i.e., by Intuitive^®^ (6.2 billion dollars), CMR surgical^®^ (450 million dollars), or Medtronic^®^ surgical innovations (1.5 billion dollars).

The RLS reports have described a refinement in progressively complex procedures, while showing results financially comparable to open resections [2], including major, anatomic, donor, and complex liver resections, supporting the hypothesis of being a reproducible, safe approach, with an increasing technical ceiling [3], and a faster learning curve compared to the laparoscopic approach [4], which may allow moving to robotic from the open approach with no need for a previous full laparoscopic learning curve.

In the pan-European survey on the implementation of robotic and laparoscopic minimally invasive liver surgery [5], only 28% of surgeons surveyed reported performing major procedures, and 29% minor, and up to 46% described their method of liver transection with the use of bipolar forceps, omitting the CUSA. Although 30% of surgeons stated they prefer robotic surgery, they expect an increasing implementation of RLS in the future, admitting it could be more expensive than LLS.

Several consensus has giving the LLS a wide acceptance and a high recommendation degree, such as the Louisville [6] and Morioka declarations [7], the International Liver Laparoscopy Society [8], the Southampton Guidelines [9], or the Consensus Guidelines [10], while classifying the RLS as non-inferior approach, mainly due to the lack of high-quality evidence.

Despite this, it is accepted that the RLS is superior by providing an expanded three-dimensional 3D stereo vision, ergonomic station, very good bipolar and monopolar energy, enhanced flexibility (thanks to the 7 degrees of movement of the robotic arms), and tremor filter (useful to perform fine dissection of vital structures and sutures in narrow space), helping to overcome the shortcomings experienced in conventional LLS.

The technical developments of RLS had boosted its use in the clinical practice, as well as the international series reports [11, 12]. The international consensus statement on RLS also contributed to its standardization [13]. Furthermore, some important issues, such as the cost-effectivity or cost-efficiency results remain controversial.

Methodologically, RLS has been developed from the LLS lessons learned, such as the caudal approach for the hilar plate [14], the Laennec capsule for the “liver gates” [15], and the “cone units” [16], improving the anatomical precise and parenchyma-preserving resections.

Technically, the robot introduces several innovative features that favors the intraoperative navigation, such as the integrated in-console vision of the intraoperative ultrasound, and the simple switch to indocyanine green (ICG) vision for the negative and positive parenchyma staining [17, 18], but also the virtual 3D model assistance [19], and identification algorithms [20]. That is why, the RLS is currently in stage 2a of the IDEAL development framework of surgical innovations [21] (equivalent to pioneer surgeon), while the LLS is in stage 3, close to stage 4 (“early adopter” for many centers, but being established practice in others).

Notwithstanding, the available instruments for liver parenchymal transection (LPT) are limited, as the robot do not offer the tools former established for open and laparoscopic LPT, such as the cavitronic dissector, the harmonic sealer, or the radio-frequency coagulator. That is why the absence of a systematized technique has led to the development of several options for LPT, which in many cases have become standard in each institution.

This variability in the LPT technique is the origin of a controversial widespread heterogeneity, that significantly limits the overall analysis of the technical ceiling of the robotic approach, making it difficult to assess the cost associated to liver resections.

Robotic LPT can be performed under three modalities, regarding the current state-of-the-art:

- Robotic Assisted, when the scrubbed assistant surgeon transects the liver parenchyma with a laparoscopic tool that is foreigner to the robot system, but under robotic assistance, i.e., laparoscopic cavitron ultrasonic surgical aspirator [22–25], or waterjet [26].
- Totally Robotic, using advanced robotic tools, such as the Vessel Sealer [27], the Harmonic Scalpel [28–30], the SLiC saline-linked electrocautery [31], or the Synchroseal [32].
- Real Robotic, with no use of laparoscopic or robotic advanced tools, using only plugged bipolar fenestrated forceps, bipolar Maryland forceps, monopolar curved scissors, or monopolar permanent cautery spatula.

As no robotic platform includes the specific standardized tooling for LLS, it can be stated that the minimal common LPT technique option has to be based upon the use of the tools offered as standard by the platform (basically forceps and scissors), along with the selective use of the bipolar and monopolar energy, respectively, in order to progress into the transection plane through small steps we may call “microfracture-coagulation” (MFC).

The first reports of the real robotic LPT were described as “crush-clamp” technique variants, with the use of bipolar energy devices [33–35], but the MFC method has not been yet systematized.

This technique is systematically used in all RLS cases at our institution since 2018 [36]. The series (Table 1) includes 131 robotic liver resections for 138 lesions, performed in 123 patients with the Da Vinci Xi Surgical System, consecutively collected between April 2018 and October 2023. Patients were aged 63.7 (20–82) years, mainly men (53.7%), with median BMI 27.7, and median Charlson comorbidity index 7.1. Main indication was malignancy (74.8%). Surgical resections were predominantly anatomical: 83 cases (67.5%), including major hepatectomies (10.7%) and two-stage hepatectomies (2 ALPPS cases). There were 66 cases of lesions in posterior segments 6,7,8 (42.5%), considered difficult in LLS. The mean operative time was 217.6 min, with a Pringle hilar clamping time of 50.9 (17–123) min. The mean blood loss was 168.1 ml, and 4 patients received perioperative transfusion. The median total hospital stay was 4.2 days. Morbidity before 90 days postoperatively Clavien-Dindo ≥ grade 3 in 6 cases (4.9%), with 3 ISGLS B/C bile leaks, and 3 cases of conversion: 1 to laparoscopy (irreversible energy failure) and 2 to open surgery (adhesion syndrome, and hidden bleeding point check after procedure). There was 1 case of re-intervention (laparoscopic intestinal lesion prior to docking), and 1 case of mortality (ISGLS grade 3 irreversible postoperative liver failure after anatomical resection of segment 8 in a Child B cirrhotic patient).

**Table 1:** Sample series. Baseline characteristics and perioperative details

Descriptive data	RLS (n = 123)
Preoperative baseline characteristics	
Age, year, median (IQR)	63.7 (20–82)
Female, *n*º (%)	57 (46.3)
BMI, kg/m^2^, median (IQR)	27.7 (18.1–41.4)
ASA, *n*º (%) I–II	51 (41.5)
ASA, *n*º (%) III–IV	72 (58.5)
CCI, median (IQR)	7.1 (1–12)
Preoperative diagnosis	
Malignant, *n*º (%)	92 (74.8)
CRCM	50 (40.6)
NCRCM	6 (4.9)
HCC	28 (22.8)
IHCC	7 (5.7)
GBC	1 (0.8)
Benign, *n*º (%)	31 (25.2)
Intraoperative	
Resections, nº	131
Lesions, nº	138
Lesions in posterior segments (6,7,8), *n*º (%)	66 (53.7)
Size in mm, median (IQR)	39.9 (4–170)
Major liver resections, *n*º (%)	13 (10.7)
Right hemihepatectomy, *n*º (%)	5 (4.1)
Left hemihepatectomy, *n*º (%)	8 (6.5)
Anatomic minor liver resections, *n*º (%)	70 (56.9)
Left lateral sectorectomy, *n*º (%)	26 (21.1)
Right posterior sectorectomy, *n*º (%)	3 (2.4)
Central hepatectomy, *n*º (%)	2 (1.6)
Segmentectomy, *n*º (%)	39 (31.7)
Parenchyma-sparing liver resections	40 (32.5)
Operative time, median (IQR)	217.6 (120–390)
Pringle hilar clamping time, median (IQR)	50.9 (17–123)
Conversions, *n*º (%)	3 (2.4)
Transfusions, *n*º (%)	4 (3.3)
Blood loss in ml, median (IQR)	168.1 (100–900)
R0 oncological free margin, *n*º (%)	78 (90.1)
Postoperative	
Length of hospital stay in days, median (IQR)	4.2 (2–14)
Reintervention, *n*º (%)	1 (0.8)
Severe morbidity (Clavien-Dindo ≥ grade 3), *n*º (%)	6 (4.9)
ISGLS Bile leakage grade B/C, *n*º (%)	3 (2.4)
Mortality < 90 days postoperative, *n*º (%)	1 (0.8)

*RLS* robotic liver surgery, *ASA* american society of anesthesiologists physical status classification system score, *BMI* body mass index, *CCI* charlson comorbidity index, *CRCM* colo-rectal cancer metastases, *NCRCM* non colo-rectal cancer metastases, *HCC* hepato-cellular carcinoma, *IHCC* intra-hepatic cholangio-carcinoma, *GBC* gallbladder cancer, *ISGLS* international study group of liver surgery

### Surgical technique

#### Indications

MFC is indicated in any type of robotic liver resection, from minor to major, and from parenchyma-sparing to enlarged anatomical liver resections, including complete piggy-back/ hanging maneuver and two-stage liver resection.

#### Technical description

MFC for real robotic LPT can be defined by the simultaneous and synchronized use of the EndoWrist bipolar fenestrated forceps and the EndoWrist monopolar curved scissors, both plugged into the integrated ERBE VIO dV 2.0 generator cut and coagulation (effect 6), usually under extracorporeal Pringle hilar clamping.

**Fig. 1:**
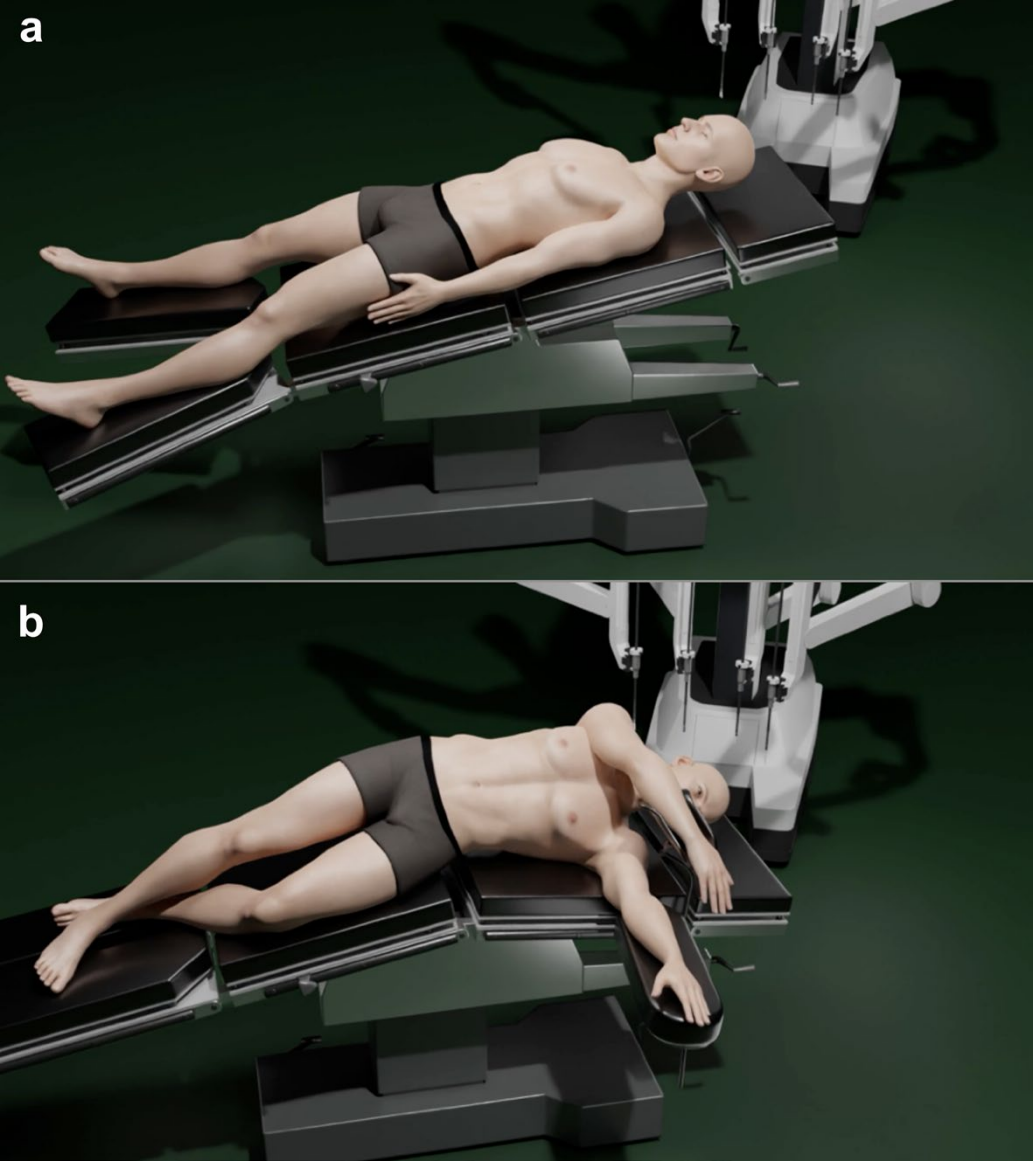
Patient positioning. (**a**) Supine decubitus open-legged French position with 8° anti-Trendelemburg for anterior lesions. (**b**) Left decubitus for right posterior lesions

The patient positioning (Fig. 1) is supine decubitus open-legged, with the arms closed, with 8° anti-Trendelemburg, above body vacuum mattress. The position may be modified with the integrated table position at will during the procedure, although left decubitus may be used for true right posterior lesions resections.

**Fig. 2:**
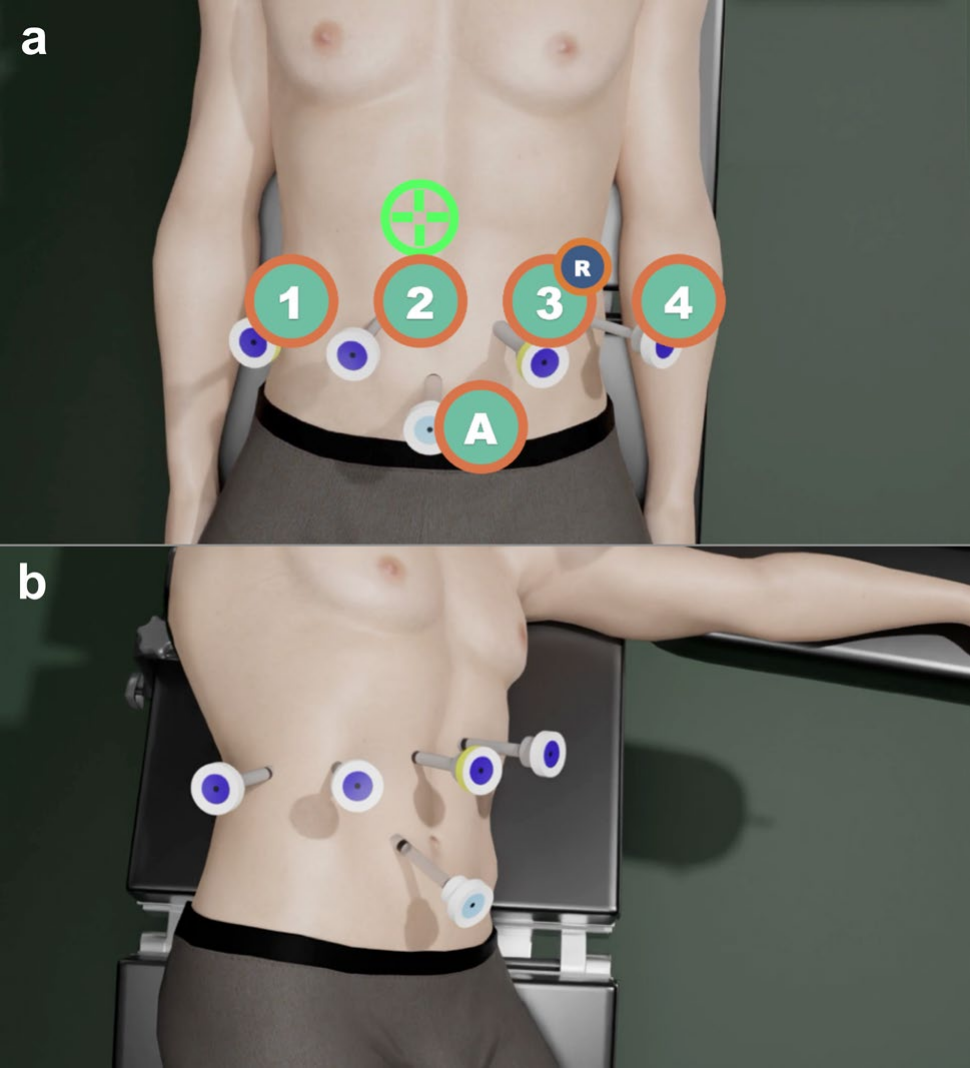
Trocar placement. (**a**) Supine decubitus for anterior lesions. Trocar placement above the umbilicus (1: Fenestrated forceps port, 2: Pointer and camera port, 3: Curved scissors trocar, R: 12–8 mm reducer cannula, A: Assistant 12 mm laparoscopic trocar); (**b**): Left decubitus for right posterior lesions. Subcostal trocar placement

The 4-trocar placement (Fig. 2) follows a horizontal line above the umbilicus, leaving bipolar forceps left to the camera trocar, and monopolar scissors right to it, leaving the fourth trocar free for liver mobilization and traction. Depending on the body mass index, the fourth trocar may be placed slightly upper from the trocar baseline. The right tool trocar (usually nº 3, is a 12 mm trocar with 12–8 mm reducer cannula to admit EndoWrist SureForm 60 mm and 45 mm curved-tip endostaplers). One assistant trocar may be placed below, 7 cm equidistant from the camera trocar and the curved scissors trocar, to irrigate/suction, or to provide material supply, as gauze or stitches, as needed. The trocar placement for left decubitus follows the same disposition, but leaving the subcostal anterior axillary point for trocar 2 pointer. The hilar Pringle clamping is extracorporeal with a Rommel tourniquet using a 24FR Nelaton catheter through a 5 mm left incision for right liver lobe lesions, but right for left lobe lesions.

The Glisson capsule is incised with the curved monopolar scissors, making a 1–2 cm fence along the desired transection line, once the navigation tools are checked in-console (i.e., intraoperative ultrasound, ICG dye staining, or 3D model consultation).

**Fig. 3:**
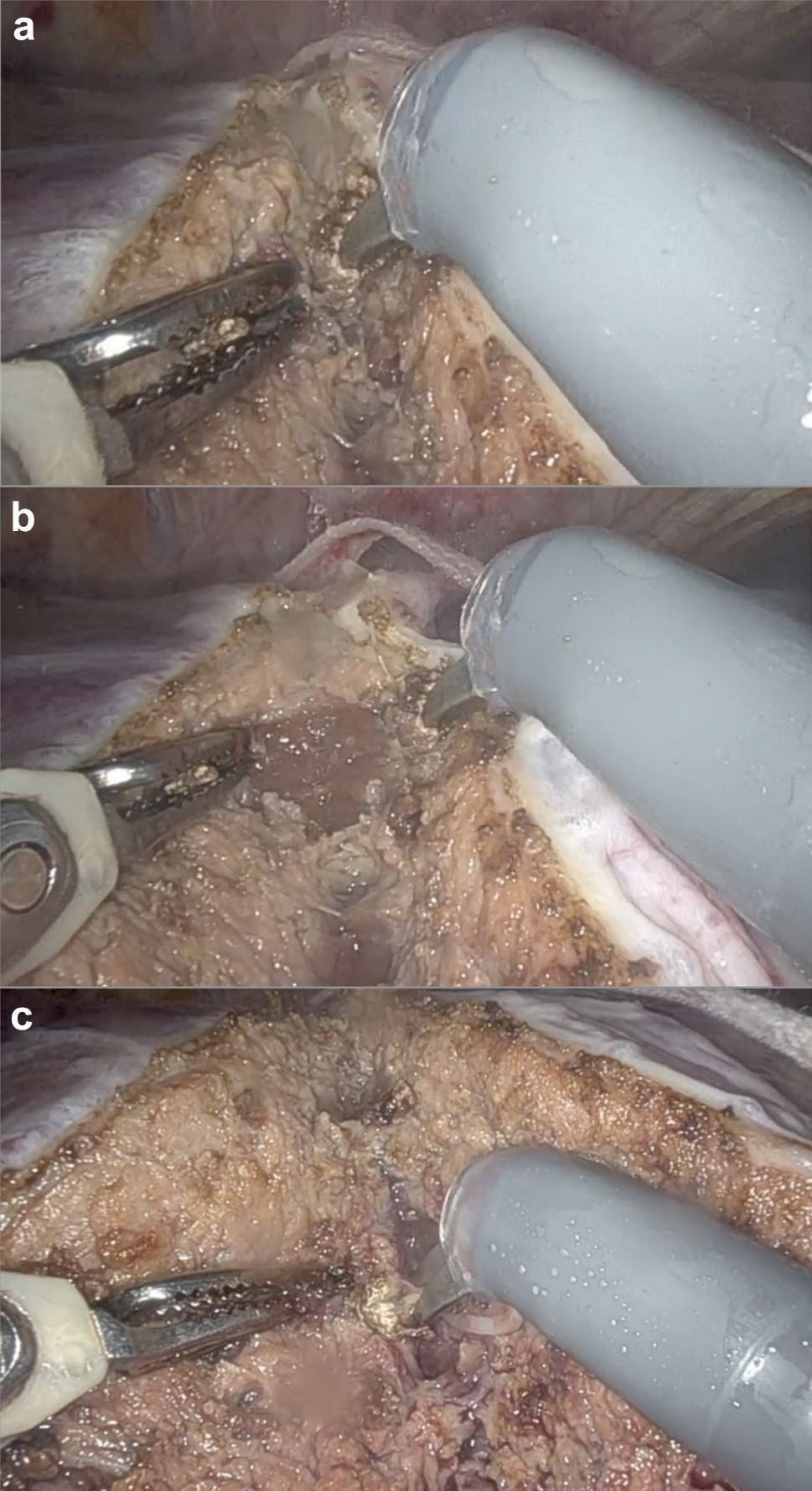
Microfracture-coagulation steps. (**a**): First step cold progression. Initial position; (**b**): First step cold progression. Final position after microfracture; (**c**): Bipolar and monopolar energy coagulation

The method of progression during the LPT is subdivided into three consecutive steps, as follows (Fig. 3):

1-First step: Cold progression, starting in contact with the transection cutting surface, where the separation of the tooltips fractures the parenchyma towards deep, thus carefully revealing the anatomy of the communicating vessels and the 3rd order glissonian and main hepatic vein branches. The EndoWrist monopolar curved scissors may dissect the vessel and surround it 360º to obtain a security stump after cutting.

2-Second step: Bipolar energy application, in which the bipolar forceps coagulates the selected vessel (up to 5 mm) by diathermy, before cutting it with monopolar energy with the scissors.

3-Third step: Monopolar energy application, in which the monopolar curved scissors coagulates the new transection frontline before proceeding to repeat the series.

Vessels up to 15 mm may be isolated by cold dissection in a segment wide enough to apply medium-large locked clips with the robotic applier, prior to section it with scissors, while first and second order glissonean pedicles may be identified without being injured, dissected, surrounded with a loop with the wristed forceps, and lift it up, thus allowing the progress of the wristed robotic endostapler for mechanical transection, with SureForm wristed da Vinci blue reload 45–60 staplers. Main hepatic veins root dissection may be transparenchymatous during major hepatectomies, and transected with tipped 35 white reload stapler.

**Fig. 4:**
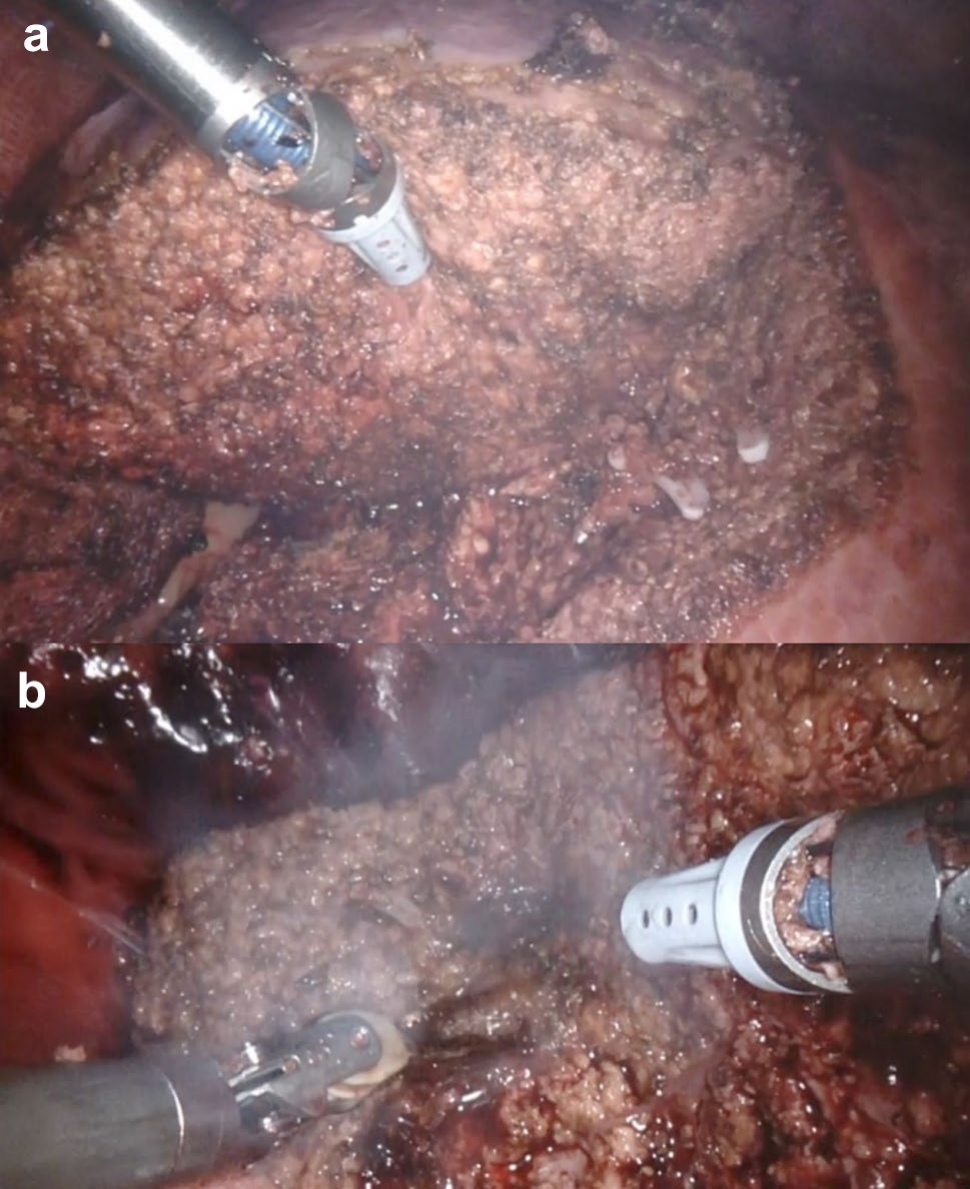
Microfracture-coagulation. (**a**): Final transection check. (**b**): Superficial bipolar coagulation is applied

The final transection surface is checked at the end of the procedure (Fig. 4). This revision is usually done after releasing the hilar clamp, by applying gauze onto the transection surface, and then removing it rolling over, uncovering one by one the potential oozing points, so superficial bipolar coagulation can be applied selectively, avoiding monopolar coagulation that could leave ischemic bedsores areas below, and eventually be the origin of potential bilomas or hematomas.

## Discussion

The pure robotic systematic to perform the LPT is described, that we refer to as MFC. This technique can be commonly used for the robotic platforms, with no need for advanced tools or laparoscopic instruments, as the minimally most effective methodology for LPT. During the LPT, only the platform’s own tools are used, without using advanced energy instruments, neither compatible with the robot, nor external laparoscopic tools through an accessory port, just as a real robotic technique.

It allows the precise and fine dissection of critical structures in order to achieve a safe transection, minimizing the possibility of hemorrhagic events, thus avoiding complications, while maximizing the identification of minor bile leaks, so they can be early identified and primarily sutured or clipped.

The use of this parenchymal transection methodology obtains a bloodless hepatic surgical plane, equivalent to that obtained by laparoscopy. Paradoxically, despite being based on the use of bipolar and monopolar energy, progress in transection is mainly cold, through small microfracture steps, sparing the liver parenchyma itself, being high preservative for it at the same time, freeing so the glissonean structures from the limiting hepatic plate, and the main hepatic veins from the vascular adventitious layer of collagen and elastic fibers, so precise bipolar coagulation may be applied at will. Notwithstanding, the final cutting parenchymatous surface gets only discretionary coagulated, so hemostasis must be checked at the end of the resection.

### Rationale

A major justification for the systematic use of MFC is the optimization of the robotic platform. Through the systematic use of the standard tools, it is possible to reduce costs to the minimum, while pushing up its technical possibilities, thus eliminating the inherent variability between the different methods, as well as focusing on the technical improvements, thus defining the technical ceiling of such an approach. Another major justification is the possibility of systematizing the LPT method, and potentially obtaining more standardized series, so making the results comparable, but also eliminating the confusion bias associated with the intra-institution evolution, or eventual change of method within the same series during the learning curve, maximizing its interpretation and the comparison between the different series, thus facilitating this way the meta-analysis performance.

### Advantages and disadvantages in the context of other techniques and published studies

Since there is no single system for LPT, it can be inferred that the different groups that have reported alternative techniques, also validating them as standard within each center, have not reported technical limitations associated with each one, so it can be difficult to assess the real advantage between the different methods, as well as their possible adoption at an international level.

As reported in our series [36], MFC may require slightly longer hilar clamping timings, with no statistically significant differences in the operating time, but associated with minor blood loss and transfusion rate, as well as wider free oncological margins. It has to be noted that during its use in advanced resections, one important limitation is the possibility of compromising the free oncological margin when approaching the lesion as the transection plane progresses, due to the mass effect that every space-occupying lesion produces on the surrounding liver parenchyma. To avoid this, it is advisable to advance in small microfracture steps, reevaluating in each one the transection plane in relation to the distance of the lesion and the desired lesion-free margin. On the other hand, the observation of this caution is inherently aligned with the prevention of the appearance of bleeding points, thus obtaining a more bloodless plane throughout the transection, helping to the early detection of fine structures.

## Conclusion

MFC is a precise real robotic method for the LPT, using the standard tooling of the robotic platforms, mainly fenestrated forceps and curved scissors, by small cold microfracture steps, combined with the application of bipolar and monopolar energy. It is reproducible and safe, indicated in all types of robotic liver resections, and comparable. It obtains a bloodless transection plane, analogous to that obtained by laparoscopy, optimizing the precise dissection of fine structures, and maximizing the early control of possible bile leaks or bleeding. Its use as a default liver transection method in robotic approach should be considered.

## Data Availability

As no systematization exists over the robotic LPT, and data are not truly comparable, the authors leave the open question for the scientific community. The main references over the issue are provided.

## References

[1] Emmen AMLH, Görgec B, Zwart MJW, Daams F, Erdmann J, Festen S, Gouma DJ, van Gulik TM, van Hilst J, Kazemier G, Lof S, Sussenbach SI, Tanis PJ, Zonderhuis BM, Busch OR, Swijnenburg RJ, Besselink MG, for HPB-Amsterdam, (2023) Impact of shifting from laparoscopic to robotic surgery during 600 minimally invasive pancreatic and liver resections. Surg Endosc 37(4):2659–2672. 10.1007/s00464-022-09735-4 36401105 10.1007/s00464-022-09735-4 PMC10082117

[2] Daskalaki D, Gonzalez-Heredia R, Brown M, Bianco FM, Tzvetanov I, Davis M, Kim J, Benedetti E, Giulianotti PC (2017) Financial impact of the robotic approach in liver surgery: a comparative study of clinical outcomes and costs between the robotic and open technique in a single institution. J Laparoendosc Adv Surg Tech A 27(4):375–382. 10.1089/lap.2016.0576 28186429 10.1089/lap.2016.0576 PMC5397272

[3] Chong CC, Fuks D, Lee KF, International Robotic and Laparoscopic Liver Resection study group investigators et al (2022) Propensity score-matched analysis comparing robotic and laparoscopic right and extended right hepatectomy. JAMA Surg. 10.1001/jamasurg.2022.0161 35262660 10.1001/jamasurg.2022.0161 PMC8908223

[4] Gall TMH, Alrawashdeh W, Soomro N, White S, Jiao LR (2020) Shortening surgical training through robotics: randomized clinical trial of laparoscopic versus robotic surgical learning curves. BJS Open 4(6):1100–1108. 10.1002/bjs5.50353 33052038 10.1002/bjs5.50353 PMC7709379

[5] Zwart MJW, Görgec B, Arabiyat A, Nota CLM, van der Poel MJ, Fichtinger RS, Berrevoet F, van Dam RM, Aldrighetti L, Fuks D, Hoti E, Edwin B, Besselink MG, Abu Hilal M, Hagendoorn J, Swijnenburg RJ, Dutch Liver Collaborative Group and E-AHPBA Innovation Development Committee (2021) Pan-European survey on the implementation of robotic and laparoscopic minimally invasive liver surgery. HPB. 10.1016/j.hpb.2021.08.939 34772622 10.1016/j.hpb.2021.08.939

[6] Buell JF, Cherqui D, Geller DA, World Consensus Conference on Laparoscopic Surgery et al (2008) The international position on laparoscopic liver surgery: the louisville statement. Ann Surg 250(5):825–830. 10.1097/sla.0b013e3181b3b2d8 10.1097/sla.0b013e3181b3b2d8 19916210

[7] Wakabayashi G, Cherqui D, Geller DA et al (2015) Recommendations for laparoscopic liver resection: a report from the second international consensus conference held in Morioka. Ann Surg 261(4):619–629. 10.1097/SLA.0000000000001184 25742461 10.1097/SLA.0000000000001184

[8] Cherqui D, Wakabayashi G, Geller DA, Buell JF, Han HS, Soubrane O, O’Rourke N, International Laparoscopic Liver Society (2016) The need for organization of laparoscopic liver resection. J Hepatobiliary Pancreat Sci 23(11):665–667. 10.1002/jhbp.401 27770492 10.1002/jhbp.401

[9] Abu Hilal M, Aldrighetti L, Dagher I et al (2018) The southampton consensus guidelines for laparoscopic liver surgery: from indication to implementation. Ann Surg 268(1):11–18. 10.1097/SLA.0000000000002524 29064908 10.1097/SLA.0000000000002524

[10] Gotohda N, Cherqui D, Geller DA et al (2021) Expert consensus guidelines: how to safely perform minimally invasive anatomic liver resection. J Hepatobiliary Pancreat Sci. 10.1002/jhbp.1079 34779150 10.1002/jhbp.1079

[11] Dugan MM, Sucandy I, Ross SB, Crespo K, Syblis C, Alogaidi M, Rosemurgy A (2023) Analysis of survival outcomes following robotic hepatectomy for malignant liver diseases. Am J Surg. 10.1016/j.amjsurg.2023.10.034 37880028 10.1016/j.amjsurg.2023.10.034

[12] McCarron F, Cochran A, Ricker A, Mantha R, Driedger M, Beckman M, Vrochides D, Martinie J (2023) 10 years, 100 robotic major hepatectomies: a single-center experience. Surg Endosc. 10.1007/s00464-023-10459-2 37845533 10.1007/s00464-023-10459-2

[13] Liu R, Abu Hilal M, Wakabayashi G et al (2013) International experts consensus guidelines on robotic liver resection in 2023. World J Gastroenterol 29(32):4815–4830. 10.3748/wjg.v29.i32.4815 10.3748/wjg.v29.i32.4815 PMC10494765 37701136

[14] Tomishige H, Morise Z, Kawabe N, Nagata H, Ohshima H, Kawase J, Arakawa S, Yoshida R, Isetani M (2013) Caudal approach to pure laparoscopic posterior sectionectomy under the laparoscopy-specific view. World J Gastrointest Surg 5(6):173–177. 10.4240/wjgs.v5.i6.173 23977419 10.4240/wjgs.v5.i6.173 PMC3750128

[15] Sugioka A, Kato Y, Tanahashi Y (2017) Systematic extrahepatic glissonean pedicle isolation for anatomical liver resection based on Laennec’s capsule: proposal of a novel comprehensive surgical anatomy of the liver. J Hepatobiliary Pancreat Sci 24(1):17–23. 10.1002/jhbp.410 28156078 10.1002/jhbp.410 PMC5299460

[16] Takasaki K (1998) Hepatic resection using glissonean pedicle transection. Nihon Geka Gakkai Zasshi 99(4):245–250 9642694

[17] Nishino H, Hatano E, Seo S et al (2018) Real-time navigation for liver surgery using projection mapping with indocyanine green fluorescence: development of the novel medical imaging projection system. Ann Surg 267(6):1134–1140. 10.1097/SLA.0000000000002172 28181939 10.1097/SLA.0000000000002172

[18] Sucandy I, Luberice K, Lippert T, Castro M, Krill E, Ross S, Rosemurgy A (2020) Robotic major hepatectomy: an institutional experience and clinical outcomes. Ann Surg Oncol 27(13):4970–4979. 10.1245/s10434-020-08845-4 32661848 10.1245/s10434-020-08845-4

[19] Huber T, Tripke V, Baumgart J, Bartsch F, Schulze A, Weber S, Heinrich S, Lang H (2023) Computer-assisted intraoperative 3D-navigation for liver surgery: a prospective randomized-controlled pilot study. Ann Transl Med. 11(10):346–346. 10.21037/atm-22-5489 37675318 10.21037/atm-22-5489 PMC10477660

[20] Kaoukabani G, Gokcal F, Fanta A, Liu X, Shields M, Stricklin C, Friedman A, Kudsi OY (2023) A multifactorial evaluation of objective performance indicators and video analysis in the context of case complexity and clinical outcomes in robotic-assisted cholecystectomy. Surg Endosc 37(11):8540–8551. 10.1007/s00464-023-10432-z 37789179 10.1007/s00464-023-10432-z

[21] Cook JA, McCulloch P, Blazeby JM, Beard DJ, Marinac-Dabic D, Sedrakyan A, IDEAL Group (2013) IDEAL framework for surgical innovation 3: randomised controlled trials in the assessment stage and evaluations in the long term study stage. BMJ 18(346):f2820. 10.1136/bmj.f2820 10.1136/bmj.f2820 PMC3685513 23778425

[22] Wakabayashi G, Sasaki A, Nishizuka S, Furukawa T, Kitajima M (2011) Our initial experience with robotic hepato-biliary-pancreatic surgery. J Hepatobiliary Pancreat Sci 18(4):481–487. 10.1007/s00534-011-0388-3 21487755 10.1007/s00534-011-0388-3

[23] Ji WB, Wang HG, Zhao ZM, Duan WD, Lu F, Dong JH (2011) Robotic-assisted laparoscopic anatomic hepatectomy in China: initial experience. Ann Surg 253(2):342–348. 10.1097/SLA.0b013e3181ff4601 21135692 10.1097/SLA.0b013e3181ff4601

[24] Kam JH, Goh BK, Chan CY, Wong JS, Lee SY, Cheow PC, Chung AY, Ooi LL (2016) Robotic hepatectomy: initial experience of a single institution in Singapore. Singapore Med J 57(4):209–214. 10.11622/smedj.2016024 26843059 10.11622/smedj.2016024 PMC4853489

[25] Hawksworth J, Radkani P, Nguyen B et al (2022) Improving safety of robotic major hepatectomy with extrahepatic inflow control and laparoscopic CUSA parenchymal transection: technical description and initial experience. Surg Endosc 36(5):3270–3276. 10.1007/s00464-021-08639-z 34370124 10.1007/s00464-021-08639-z

[26] Perrakis A, Rahimli M, Gumbs AA, Negrini V, Andric M, Stockheim J, Wex C, Lorenz E, Arend J, Franz M, Croner RS (2021) Three-device (3D) technique for liver parenchyma dissection in robotic liver surgery. J Clin Med 10(22):5265. 10.3390/jcm10225265 34830547 10.3390/jcm10225265 PMC8653962

[27] Calin ML, Sadiq A, Arevalo G, Fuentes R, Flanders VL, Gupta N, Nasri B, Singh K (2016) The first case report of robotic multivisceral resection for synchronous liver metastasis from pancreatic neuroendocrine tumor: a case report and literature review. J Laparoendosc Adv Surg Tech A 26(10):816–824. 10.1089/lap.2016.0342 27454160 10.1089/lap.2016.0342

[28] Goja S, Singh MK, Vohra V, Soin AS (2015) Robotic left hepatectomy: a case report (first reported case of robotic hepatectomy in India). Indian J Surg 77(4):338–340. 10.1007/s12262-015-1307-7 26702246 10.1007/s12262-015-1307-7 PMC4688264

[29] Quijano Y, Vicente E, Ielpo B et al (2016) Hepatobilio-pancreatic robotic surgery: initial experience from a single center institute. J Robot Surg 11(3):355–365. 10.1007/s11701-016-0663-z 28039607 10.1007/s11701-016-0663-z

[30] Choi GH, Chong JU, Han DH, Choi JS, Lee WJ (2017) Robotic hepatectomy: the Korean experience and perspective. Hepatobiliary Surg Nutr 6(4):230–238. 10.21037/hbsn.2017.01.14 28848745 10.21037/hbsn.2017.01.14 PMC5554764

[31] Fujikawa T, Uemoto Y, Matsuoka T, Kajiwara M (2022) Novel liver parenchymal transection technique using saline-linked monopolar cautery scissors (slic-scissors) in robotic liver resection. Cureus. 14(8):e28118. 10.7759/cureus.28118 36158368 10.7759/cureus.28118 PMC9484006

[32] Görgec B, Zwart M, Nota CL et al (2022) Implementation and outcome of robotic liver surgery in the netherlands: a nationwide analysis. Ann Surg 277(6):e1269–e1277. 10.1097/SLA.0000000000005600 35848742 10.1097/SLA.0000000000005600 PMC10174096

[33] White MG, Tzeng CW, Ikoma N, Chun YS, Aloia TA, Vauthey JN, Cao HST (2021) Robotic partial segment VIII resection. Ann Surg Oncol 28(3):1513. 10.1245/s10434-020-08999-1 32761429 10.1245/s10434-020-08999-1 PMC8601113

[34] Newton AD, Newhook TE, Ikoma N et al (2022) Robotic extended right hepatectomy for colorectal liver metastasis. Ann Surg Oncol 29:8455. 10.1245/s10434-022-12493-1 36112251 10.1245/s10434-022-12493-1

[35] Cugat Andorrà E, Cremades Perez M, Navinés López J et al (2022) Challenge and future of liver and pancreatic robotic surgery. Analysis of 64 cases in a specialized unit. Cir Esp (Engl Ed) 100(3):154–160. 10.1016/j.cireng.2022.02.012 35221241 10.1016/j.cireng.2022.02.012

[36] Navinés-López J, Pardo Aranda F, Cremades Pérez M, Espin Álvarez F, Zárate Pinedo A, Sentí Farrarons S, Galofré Recasens M, Cugat Andorrà E (2023) Robotic liver surgery: A new reality. Descriptive analysis of 220 cases of minimally invasive liver surgery in 182 patients. Cir Esp (Engl Ed). 10.1016/j.cireng.2023.04.013 37105365 10.1016/j.cireng.2023.04.013

